# Maladaptive perfectionism can explain the inverse relationship between dispositional mindfulness and procrastination

**DOI:** 10.1371/journal.pone.0318845

**Published:** 2025-02-12

**Authors:** Gozde Ibili, Jeremy John Tree, Yılmaz Orhun Gurluk

**Affiliations:** 1 School of Psychology, Swansea University, Swansea, United Kingdom; 2 Psychometrics, Psychology Department, Ege University, Izmir, Turkey; Kazimierz Wielki University in Bydgoszcz: Uniwersytet Kazimierza Wielkiego w Bydgoszczy, POLAND

## Abstract

Given the widespread occurrence of procrastination and its adverse association with well-being, investigating the individual variables that influence procrastination is a crucial matter. Previous research has identified dispositional mindfulness to be negatively associated with procrastination, but the underlying mechanisms driving this relationship remain unclear. In this study, the aim was to investigate whether the inverse relationship between dispositional mindfulness and procrastination could be explained by the mediating roles of trait anxiety and maladaptive perfectionism. In a cross-sectional survey, 126 participants (aged 18–33) completed the 15-Item Five-Facet Mindfulness Questionnaire, the State-Trait Anxiety Inventory-Trait Form, the Almost Perfect Scale-Revised, and the Pure Procrastination Scale. A parallel mediation model was tested to investigate the mediating role of self-reported maladaptive perfectionism and trait anxiety to explain the relationship between dispositional mindfulness and procrastination with a bootstrapped multivariate technique. The results revealed that maladaptive perfectionism significantly mediated the relationship between mindfulness and procrastination -indicating that dispositional mindfulness has a significant indirect effect on procrastination via decreased levels of maladaptive perfectionism. It was determined that 15% of the variance in procrastination was significantly explained by this model (*R*^*2*^ = .15, β =  −.39, *B* =  −.83, 95% CI =  [−1.18, −.48], *p* < .001]. As the study highlights the importance of maladaptive perfectionism to explain the link between mindfulness and procrastination, we suggest that future research could investigate the influence of mindfulness on procrastination via mindfulness-based interventions, and include measurements of both trait anxiety and maladaptive perfectionism across longitudinal or experimental designs to unpack causality with respect to our pattern of observed findings.

## Introduction

Procrastination is a complex phenomenon that is related to existing personality characteristics, and adverse consequences including psychiatric symptoms [1]. According to DeSimone as cited in Ferrari and colleagues [1], the term *procrastination* means to postpone until another day. Even though the conceptualization of procrastination differs historically, one steady component of all conceptualizations is postponing or delaying a task [2]. Procrastination is described as not only an issue of time management, but rather a complex interaction of behavioural, cognitive, and emotional components [3,4]. The widespread definition of procrastination comes from Steel [2] who defines procrastination as ‘to voluntarily delay an intended course of action despite expecting to be worse off for the delay.’ (p.66), and indicates it as a form of self-regulatory failure.

It is suggested that everyone procrastinates to some extent, but some people procrastinate more than others [5–7]. The prevalence of procrastination is suggested to be especially striking in student populations. The research by Ellis and Knaus as cited in Steel [2], underscores that up to 95% of students engage in procrastination occasionally. Moreover according to the study of Hill and colleagues as cited in Ferrari and colleagues [1], more than half of students identify themselves as procrastinators when it comes to performing academic tasks. Day and colleagues [6] further revealed that more than 30% of students are severe procrastinators who engage in problematic and chronic procrastination. Similar to these findings, Klassen and colleagues [8] indicated that 25% of undergraduate students reported that procrastination affected their academic functioning greatly, and more than half of the students wished to reduce it.

Even though procrastination seems to be more common among student populations, research has shown that it is also prevalent among the general population. The prevalence of chronic procrastination in adults is estimated to be 20% regardless of gender [9]. McCown and Johnson as cited in Ferrari and colleagues [1], further showed that a quarter of subjects identified procrastination to be a significant problem in their lives. These studies highlight that the prevalence rates in the adult population seem to be substantially lower compared to student populations. However, it is important to note that research on procrastination in student populations explicitly focuses on academic procrastination or academic tasks whereas research on other adult populations tends to focus on everyday procrastination. Subsequently, we argue that it might be better not to compare students’ academic procrastination prevalence rates with adults’ general procrastination prevalences due to substantial differences in the measured construct.

Procrastination, which is a common phenomenon, comes with important costs. Procrastination has been associated with various negative health and well-being outcomes including; psychological distress [10], poorer mental health and less mental help-seeking behaviours [11], experiencing guilt and anxiety [5], lower self-esteem [12], depression and rumination [13,14], elevated stress and poorer physical health [15], delaying treatment and more visits to health-care professionals due to reported illnesses and symptoms in the long run [16,17]. In academic settings, procrastination has also been linked to poorer academic performance and lower grades [8,12,16,18–20].

Procrastination, although intensely studied in the literature, is still not thoroughly understood. Given the widespread occurrence of procrastination and its associations with adverse processes, it is vital to investigate and identify the individual variables that link to procrastination, and further explore the potential mechanisms to explain the relationship between these individual variables and procrastination.

### Procrastination as a self-regulation failure

Research supports the notion that self-regulation is a central construct in procrastination [21,22]. Hence, the Self-Regulation Theory which was initially developed by Bandura as cited in Bu and colleagues [23], has been widely used to explain procrastination.

Bandura as cited in Bu and colleagues [23], explains that self-regulation refers to thoughts, emotions, and actions that are generated by one, and it involves the process of directing and shifting both mental and physical functioning to attain personal goals. As mentioned by Zimmerman as cited in Zimmerman [24], self-regulation depends on individuals’ beliefs about themselves such as self-efficacy and self-doubt, and their emotions such as anxiety. Tice and Bratslavsky as cited in Eckert and colleagues [25], explain that some tasks might elicit undesired emotions such as anxiety, and people might avoid the task as a way to eliminate the negative emotions that come with it. This is supported by research demonstrating that the ability to modify and tolerate undesired emotions is important to overcome procrastination [25]. Before moving on to the literature review, it is important to mention a specific self-regulation theory to further clarify the rationale for the current study.

#### Cybernetic self-regulation theory.

One prominent theory on self-regulation is proposed by Carver and Scheier as cited in Carver [26]. Carver and Scheier’s Cybernetic Self-Regulation Theory places an explicit focus on emotions in goal attainment. The model emphasizes that there is a natural link between affect and behaviour through feedback loops which include four elements: an input function, a reference value, a comparator, and an output function. Within these feedback loops, it is important to highlight a process called *the expectancy-assessment process* to emphasize not only the emotional but also the cognitive component of self-regulation. The expectancy-assessment process involves the beliefs that the perceived discrepancy can be reduced. This process is especially important to clarify the importance of cognition in both self-regulation and procrastination. To explain this, perfectionism is of particular interest here as a cognitive component of self-regulation, and procrastination. Based on the works of Frost and colleagues as cited in Newman and colleagues [27], perfectionism refers to setting overly high standards, followed by excessive self-criticism. Given that perfectionism refers to excessively high standards -reference points-, a greater discrepancy between the current state and the reference point emerges which relates to the expectancy of not being able to reduce the discrepancy. According to Carver and Scheier as cited in Sirois and colleagues [28], this further leads to disengaging from the task or goal withdrawal.

As highlighted by several authors [3,4], procrastination is a multifaceted phenomenon with important emotional, cognitive, and behavioural processes underlying it. For the purposes of the current study, the focus will explicitly be on the emotional and cognitive components of procrastination building on the mentioned theories of self-regulation. The current study aims to consider mindfulness as a component of self-regulation and investigate how dispositional mindfulness could link to procrastination through cognition and affect. That is, we would expect dispositional mindfulness to be linked to other variables including anxiety and perfectionism. We will focus on perfectionism as a key cognitive component of procrastination and consider anxiety as a key affective component. The next section will provide insight into the relationships between these variables, and procrastination.

### Mindfulness and procrastination

According to Kabat-Zinn as cited in Shapiro and Schwartz [29], mindfulness involves elements of present-moment awareness with an explicit focus on acceptance of experiences, emotions, thoughts, and sensations with a nonjudgemental attitude at its core [23]. Moreover, mindfulness adds to self-regulation by explicitly directing conscious attention to experiences, thoughts, emotions, and sensations that occur in the present moment [29]. Mindfulness can be conceptualized and measured both as a state that varies within persons over time and is induced by mindfulness-based interventions, and a trait *or disposition* that varies between persons [30,31].

Dispositional *or trait* mindfulness is operationally defined as ‘the general tendency to be mindful in daily life’ [29] (p.757), and according to Kabat-Zinn as cited in Pérez-Aranda and colleagues [32], it includes elements of paying attention to the present moment with a nonjudgemental attitude and acceptance.

Previous studies have identified a negative relationship between dispositional mindfulness and procrastination [14,15,33–36] implying that individuals who are more mindful tend to procrastinate less. This is not surprising considering that self-regulation appears to be a central process in procrastination, and based on the evidence suggesting that dispositional mindfulness is associated with better self-regulation of emotions alongside improvements in psychological health through altering cognitions including negative thinking patterns [37].

In support of the research findings validating the negative relationship between dispositional mindfulness and procrastination, interventions that aim to increase mindfulness (i.e., mindfulness interventions and mindfulness-based educational packages) are found to be effective in decreasing procrastination [38,39], implying a causal effect of mindfulness on procrastination. A longitudinal study by Cheung and Ng [33], showed an inverse and bidirectional relationship between dispositional mindfulness and procrastination. The study pointed out that cultivating mindfulness is important to reduce procrastination based on the evidence showing that mindfulness predicts subsequent procrastination tendencies. Consequently, Glick and colleagues [34] have argued that being mindful in daily life could have a unique effect on procrastination.

Previous research identifies dispositional mindfulness to be inversely associated with procrastination. The first limitation of the current literature on procrastination is its explicit focus on student samples and academic procrastination. Consequently, current research on procrastination does not provide sufficient information about adult samples and everyday procrastination. The second limitation involves a gap in the literature with a lack of research investigating why or how the relationship between procrastination and dispositional mindfulness emerges. This gap in the literature raises the importance of investigating the potential *mediating* factors between dispositional mindfulness and procrastination as emphasized by many authors [33,34]. Where previous literature comes to light, there is only one study investigating the potential mediating mechanisms between dispositional mindfulness and procrastination. In the study by Gautam and colleagues [40] it was shown that anxiety mediated the relationship between dispositional mindfulness and procrastination in a college sample. In other words, higher levels of dispositional mindfulness indirectly decreased procrastination by reducing anxiety. This study also demonstrated that anxiety accounted for 23% of the variance in procrastination, making it the biggest contributor and a key mechanism in explaining procrastination. Accordingly, the current study again investigates anxiety as a mediator between dispositional mindfulness and procrastination with consideration of an additional key variable, maladaptive perfectionism.

### Trait anxiety

Spielberger [41] conceptualizes trait anxiety as “relatively stable individual differences in anxiety proneness” (p.3). On one hand, anxiety has been shown to be positively correlated with procrastination in student samples [4,35,42–45]. On the other hand, some studies have shown that anxiety does not add a significant and unique variance to the prediction of procrastination [2,46] and further concluded that anxiety should be investigated in the context of its relationship to other related variables [47]. In accordance with this situation, the focus of the current research emerges. In the current study, anxiety is considered a mediating factor between dispositional mindfulness and procrastination due to the robust evidence suggesting that mindfulness-based interventions and practices that increase dispositional mindfulness improve anxiety [37,48].

A meta-analysis by Teasdale and colleagues as cited in Khoury and colleagues [49] investigated the effectiveness of Mindfulness-Based Stress Reduction (MBSR), which enables individuals to experience their thoughts, emotions, and experiences in a nonreactive way and aims to reduce emotional reactivity. It was shown that MBSR was moderately effective in decreasing anxiety in nonclinical populations [49]. Blanck and colleagues [50] further demonstrated in their meta-analysis that stand-alone or isolated mindfulness interventions were also effective in reducing anxiety symptoms in nonclinical samples, and emphasized the importance of mindfulness exercises alone in improving anxiety. Similarly, Schumer and colleagues [51] have investigated the effectiveness of brief mindfulness interventions in improving negative affect including anxiety. Their meta-analysis only included randomized controlled trials and pointed out that these interventions were effective in reducing negative affectivity. Lastly, to explain the research evidence suggesting that mindfulness is effective in decreasing anxiety, it is important to highlight another study that was published in 2014. Lutz and colleagues [52] used the functional magnetic resonance imaging technique to investigate the neurobiological mechanisms of mindfulness. Their study showed that mindfulness intervention has led to reduced activation of brain regions that are central in emotion processing when participants were faced with negative stimuli. On the contrary, there were increased activations in the brain regions involved in emotional regulation. Importantly, they have also highlighted that this activation was negatively associated with dispositional mindfulness. That said, individuals who have increased levels of dispositional mindfulness required less resources for emotional regulation. Overall, according to Spielberger as cited in Barnes and colleagues [53], trait anxiety which is also described as perceiving more situations as threatening has a link to dysfunctional emotional regulation, and mindfulness has the potential to interrupt this by improving emotional regulation [52]. Moreover, Rahl and colleagues [54], have suggested that the acceptance and attention monitoring components of mindfulness can strengthen emotional regulation on demanding, unpleasant or boring tasks and enable sustained attention. It is important to acknowledge that mindfulness interventions that improve dispositional mindfulness are found to be effective in reducing anxiety [55], whereas mindfulness interventions that fail to increase dispositional mindfulness reveal contradicting results when it comes to anxiety reduction [56].

Subsequently, the following logical chain is proposed: theoretical accounts suggest that anxiety can be an antecedent of procrastination, and mindfulness is shown to be effective in anxiety reduction. Hence, it likely follows that mindfulness can decrease procrastination indirectly via anxiety reduction. We believe that studying anxiety might provide valuable insight into the relationship between mindfulness and procrastination.

Moreover, procrastination is a multifaceted construct [3,4] as highlighted by the relative importance of emotions and cognitions in Carver and Scheier’s Cybernetic Self-Regulation Theory as cited in Carver [26]. In accordance with this, the current study investigated maladaptive perfectionism as a potential underlying cognitive component of procrastination, and a key mechanism in explaining the relationship between mindfulness and procrastination.

### Perfectionism

Based on Frost and colleagues as cited in Newman and colleagues [27], perfectionism can be described as setting overly high standards, accompanied by excessively self-critical evaluations. Research literature around perfectionism provides valuable insights about both procrastination and mindfulness through a broad scope of published studies. Considering this, it comes as a surprise to see there are no studies to date considering perfectionism as the potential mechanism through which mindfulness links to procrastination. One reason for this could be the lack of consensus on the definition of perfectionism. It appears that the main focus of the previous research on perfectionism evolved around describing and classifying perfectionism. Regardless, we propose that perfectionism could provide invaluable insight into the relationship between dispositional mindfulness, and procrastination.

As highlighted by Slaney and colleagues [57], perfectionism is a multidimensional construct. Although perfectionism might contain positive aspects, the negative aspects are more profound. When it comes to the dimensions of perfectionism, there is no consensus in the literature with several authors identifying different dimensions. For example, Hewitt and Flett as cited in Slaney and colleagues [57] classify perfectionism into three dimensions which are socially-prescribed perfectionism, self-oriented perfectionism, and other-oriented perfectionism. Frost and colleagues as cited in Sederlund and colleagues [58], proposed another model in which they classified perfectionism as concern over mistakes, personal standards, doubts about actions, organization, parental criticism, and parental expectations. Frost and colleagues as cited in Sederlund and colleagues [58], investigated the two models and revealed two significant factor loadings. The authors have further identified self-oriented perfectionism, other-oriented perfectionism, personal standards, and organization as the *adaptive* aspects of perfectionism. The *maladaptive* aspects of perfectionism have been identified as socially-prescribed perfectionism, concern over mistakes, doubts about actions, parental criticism, and parental expectations. More recently, Rice and colleagues as cited in Slaney and colleagues [57] supported the two-factor structure of perfectionism which was again identified as maladaptive and adaptive perfectionism. Numerous studies have provided support for the two-factor structure of perfectionism to since, and have adapted this view [58–62]. Following the two-factor structure of perfectionism, Slaney and colleagues [57] have attempted to integrate existing perfectionism scales to reach a better consensus on the definition and measurement of perfectionism through extensive research and analysis. The authors described the positive -or *adaptive*- dimension of perfectionism as high personal standards and orderliness, whereas the negative -or *maladaptive*- dimension as discrepancies. In this case, discrepancies refer to the perceived difference between one’s standards and one’s actual performance. In other words, it is the perception that one does not meet their personal standards [57]. In the current study, the focus will solely be on maladaptive perfectionism. The definitions and descriptions provided by Slaney and colleagues [57] will be followed, and discrepancies will be referred to as maladaptive perfectionism.

In its different forms, maladaptive perfectionism has been found to be associated with numerous psychological variables such as psychological distress including depressive affect, anxiety, and negative automatic thoughts [63–68], lower self-esteem [65–69], and lower levels of self-compassion [70]. Additionally, it has also been linked to lower attention control, lower academic engagement, higher susceptibility to temptation, engaging more in self-defeating behaviours, and greater use of avoidant-coping strategies [60–62,65,71]. Importantly, numerous studies have found a positive association between maladaptive perfectionism, and procrastination [61,62,64,67,72–79] in line with the Cybernetic Self-Regulation Theory by Carver and Scheier as cited in Carver [26] which emphasized the importance of expectancy beliefs in linking perfectionism and procrastination.

Furthermore, correlational analyses have shown that maladaptive perfectionism has also been shown to be associated with reduced levels of dispositional mindfulness [66,69,80,81]. In support of this relationship between mindfulness and perfectionism, Motie and colleagues [39] showed that a mindfulness-based educational package that resulted in increased levels of mindfulness was also effective in reducing perfectionism, implying a causal effect of mindfulness on perfectionism. Similarly, Beck and colleagues [82] reported that engaging in mindfulness practices led to reduced levels of discrepancies in students in their experimental study. On the contrary, Olton-Weber and colleagues [81] failed to find a significant decrease in maladaptive perfectionism after a successful mindfulness intervention in adolescents. However, the participants in this study were adolescents experiencing very low levels of maladaptive perfectionism to begin with, which might have explained this null finding. In another study [83] which was conducted with students at a highly-ranked university with a culture emphasizing perfectionism and overachievement, it was seen that a mindfulness-based stress reduction program allowed individuals to take a step back from a constant striving for perfectionism through cultivating more moment-to-moment awareness and practicing nonjudgement. This allowed them to have more reasonable expectations of their performance and further combat the feelings that they always need to be perfect. Students also reported less avoidance of tasks, improved academic focus, and productivity.

Cultivating self-compassion, present moment awareness, and practicing nonjudgement through mindfulness seems to be helping individuals have more realistic expectations for themselves, and interrupt their need to strive for perfectionism [61,82,84]. Overall, it can be said that cultivating mindfulness allows individuals to let go of the constant striving to be perfect and the self-critical evaluations that accompany it through shifting their perspectives [83] and the current study proposes this could further lead to decreased procrastination.

### The current study

As indicated by the literature review, increased mindfulness can potentially ameliorate maladaptive perfectionism and anxiety, both of which we argue to be the important underlying processes linking mindfulness and procrastination. That being the case, there is, to our knowledge, no evidence in the literature to support or contradict the hypothesized relationship. As mentioned, there is only one study [40] investigating and supporting the hypothesized relationship between mindfulness and procrastination through the mediator of anxiety. The current study aims to target this gap in the literature on the potential mediating processes between dispositional mindfulness and procrastination relation by assessing the role of anxiety and maladaptive perfectionism as a means to better understand the nature of the former association.

**Fig 1:**
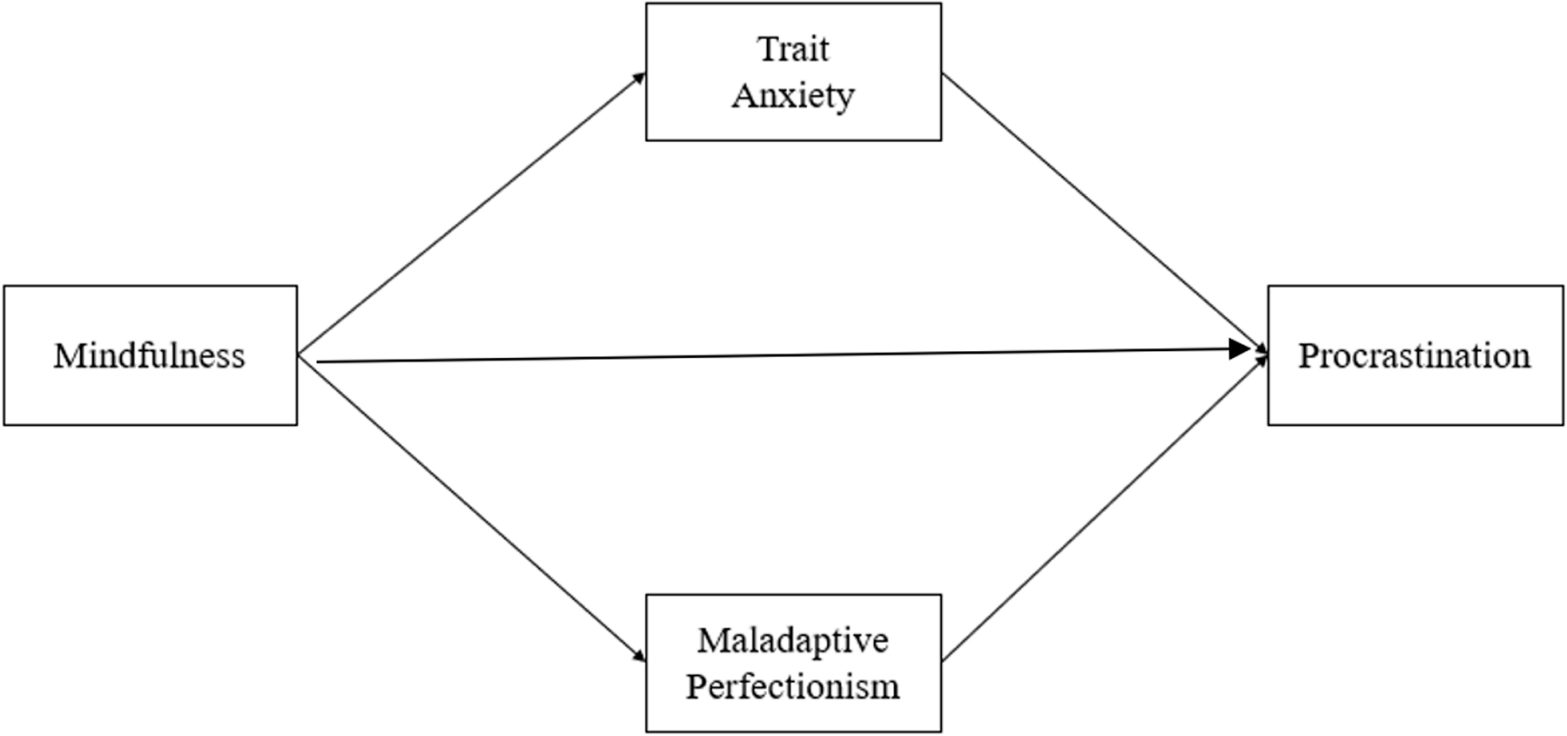
Conceptual Framework.

In line with the self-regulation theories, we initially hypothesized that dispositional mindfulness negatively predicts procrastination. Secondly, we hypothesized that the negative relationship between dispositional mindfulness and procrastination is mediated by trait anxiety and perfectionism (Fig 1). Accordingly, a parallel mediation model was tested to investigate the mediating role of self-reported maladaptive perfectionism and trait anxiety to explain the relationship between dispositional mindfulness and procrastination.

## Methods

### Participants and procedure

The total sample included 126 participants, ages ranging from 18 to 33 (*M =  22.80, SD = * 3.38). 34.1% (*N* =  43) of participants were male, and 65.9% (N =  83) were female. Participants self-identified their ethnicities as White (70.6%), Asian (17.5%), Black or African American (3.2%), Hispanic or Latino (0.8%), and other (6.3%). Two participants preferred not to disclose their ethnicity. 54% of all participants (*N* =  68) were currently undergraduate students, 20.6% (*N* =  26) were postgraduate students, and 24.6% (*N* =  31) were not students.

Participants were recruited via the Participant Pool System within Swansea University, social media advertisements, and snowballing from these. Participation was voluntary, and participants were not compensated except for participants recruited via Participant Pool. Participants who were recruited via Swansea University’s Participant Pool System were granted one credit for their participation in the study. The inclusion criteria for the current study were as follows:

1. Being 18–35 years of age,
2. Being able to provide informed consent.

The current study is a cross-sectional survey. An online questionnaire named “Assessing Individual Traits in Relation to Procrastination in Adults” was administered via Qualtrics (https:www.qualtrics.com) which is one of the main online survey distribution websites preferred by Swansea University. The final survey included validated scales of mindfulness, anxiety, perfectionism, and procrastination in addition to demographic variables including age, gender, ethnicity, and current education status. The survey was expected to take approximately 15 minutes to complete. Informed consent was gathered from all participants prior to the study. After participants completed the questionnaire, they were debriefed about the research. Data collection started after ethics approval was granted on 24/06/2022 and ended on 31/07/2022. A one-month time restriction was followed to collect data due to limited resources. Data were assessed for research purposes between 01/08/2022 and 31/08/2022. All collected data were stored in a password-protected personal computer. All data were anonymous and the researchers did not have access to information that could identify participants during or after the data collection. The research has been approved by the College of Human and Health Sciences Research Ethics Committee at Swansea University.

### Materials

#### 15-item five-facet mindfulness questionnaire (FFMQ-15) [85].

The FFMQ-15 was used to assess individuals’ dispositional mindfulness. The FFMQ-15 is a self-report questionnaire, and the items are derived from the Five-Facet Mindfulness Questionnaire (FFMQ) based on their factor loadings [86]. The questionnaire assesses five facets of mindfulness which are identified as observing, describing, acting with awareness, nonjudging of inner experience, and nonreactivity to inner experience. *Observing* refers to noticing thoughts, affect, sounds, bodily sensations, etc. *Describing* involves being able to name one’s internal experiences. *Acting with awareness* includes paying attention to what one is doing in the present moment. *Nonjudging of inner experience* relates to taking a nonjudgemental stance towards one’s thoughts, experiences, or feelings. *Nonreactivity to inner experience* refers to the tendency to allow these internal experiences, namely thoughts and emotions, to come and go [85].

The self-reported FFMQ-15 includes three items for each facet. For this study, only an overall mindfulness score which indicates the general tendency to be mindful in daily life [29,85] was of interest, and facet scores were not included in the analyses. A 5-point Likert scale is used to rate each item (1 =  *never or very rarely true,* 2 =  *rarely true,* 3 =  *sometimes true,* 4 =  *often true*, 5 =  *very often or always true*.). The scale includes seven reversed items. Before any statistical analyses, the scoring for the reversed items was corrected. After the corrections, a total score was calculated by summing up all scores. Overall mindfulness scores can vary between 15–75 with higher scores indicating higher levels of dispositional mindfulness. Example items include; “I believe some of my thoughts are abnormal or bad and I shouldn’t think that way.”, “I do jobs or tasks automatically without being aware of what I’m doing.”, and “I pay attention to sensations, such as the wind in my hair or sun on my face.”.

The previous research has indicated a good fit for the five-factor structure of mindfulness with a 90% confidence interval, supporting mindfulness as a multifaceted construct [86]. Further research supported that the five facets are indicative of an overall mindfulness construct [85,87]. Similar to the original FFMQ, the coefficient alpha values for five facets of the short version (FFMQ-15) are shown to vary from .60 to .94, indicating a largely adequate internal consistency. Internal consistency of the total score for FFMQ-15 varied from .80 to .85 [88]. Findings support that the FFMQ-15 is a valid and reliable alternative to the original FFMQ [87] and can be implemented to reduce participant burden. Since only the total score of the questionnaire was included in the current study, internal consistency of the total score FFMQ-15 was calculated and the coefficient alpha value for the current sample is found to be .68.

### State-trait anxiety inventory-trait form (STAI-T) [41].

The STAI is one of the most used and widely researched anxiety scales in the literature [89]. For this study, only the trait form of STAI was included. The trait scale of STAI aims to measure *trait anxiety*, which can be defined as the general tendency to be anxious or a rather permanent individual characteristic of one’s anxiety-proneness [41]. The STAI-T is a self-evaluation questionnaire and includes 20 items. Nine of these items are reversed. Participants are asked to rate each item on a 4-point Likert scale (1 =  *almost never,* 2 =  *sometimes*, 3 = *often*, and 4 =  *almost always*). After the corrections for scoring the reversed items, a total score is calculated by summing up all points. Scores can range from 20 to 80. Higher scores indicate higher levels of trait anxiety. Example items include; “I worry too much over something that really doesn’t matter.”, and “I am content.”.

The STAI- has shown to have high internal consistency, with coefficients varying from .86 to .92 [41]. Further research supported that the internal consistency reliability of the scale was satisfactory including a broad scope of research, and various populations [53]. Test-retest reliability of the STAI-T is shown to be adequate, ranging from .73 to .86. Concurrent validity with other anxiety scales ranged from .73 to .85 [41]. The general internal consistency-reliability coefficient alpha value for the STAI-T was found to be .91 in the current sample, indicating good internal consistency.

#### Almost perfect scale-revised (APS-R) [57].

The APS-R is a self-evaluative questionnaire that consists of 23 items. The APS-R aims to measure three dimensions of perfectionism; high standards (seven items), orderliness (four items), and discrepancy (12 items). *High standards* refers to having high expectations of oneself and holding oneself to high standards of performance. *Orderliness* involves a preference and need for neatness and organization. *Discrepancy* refers to the perceived difference between one’s standards and actual performance [57]. Participants are asked to rate each item on a 7-point Likert scale (1 = *strongly disagree*, 7 = *strongly agree*). A total score for each subscale is calculated by summing up all relevant items. Higher scores indicate higher personal standards, a stronger preference for order and neatness, and a greater discrepancy between one’s standards and one’s actual performance. Example items include; “I have high expectations for myself.” on the high standards subscale, “I like to always be organized and disciplined.” on the orderliness subscale, and “I hardly ever feel what I’ve done is good enough.” on the discrepancy subscale.

Confirmatory factor analysis revealed high internal consistency for each subscale with Cronbach’s alpha ranging from .82 to .92. Construct validity of the scale is supported by moderate to high correlations with other perfectionism scales [57]. In the current sample, the internal consistency-reliability coefficient alpha for the scale was found to be.88. Coefficient alpha values for each subscale ranged from .82 to .92.

Slaney and colleagues [57] suggested that the high standard and orderliness dimensions capture both essential and positive aspects of perfectionism. On the other hand, the discrepancy subscale is suggested to capture the essential but negative aspects of perfectionism. Research has repeatedly used the discrepancy scale solely to measure *negative* or *maladaptive* perfectionism [63,64,68,90]. Similarly, for this study, the discrepancy subscale scores were of interest which can vary from 12 to 84. In the current study, the general internal consistency-reliability coefficient alpha for the discrepancy subscale was found to be .92.

#### Pure procrastination scale (PPS) [91].

The PPS is a 12-item self-report questionnaire that aims to measure procrastination as an *irrational* or *dysfunctional delay*. It is derived from three other existing procrastination scales based on factor analysis. Unlike other scales, the PPS measures negative aspects of procrastination in all of its items. It has good reliability and construct validity. The PPS has been shown to have high internal consistency (*α* = .92). Similarly, the internal consistency-reliability coefficient alpha was found to be.91 in the current sample. The convergent validity of the scale is supported by correlations with related measures, such as the Satisfaction with Life Scale, and the Irrational Procrastination Scale [91]. Further research supports the psychometric properties of the scale, and its superiority over other existing procrastination scales [92].

In this study, participants are asked to rate each item on a 7-point Likert scale (1 = *strongly disagree,* 7 = *strongly agree).* A total score was calculated by adding together all item scores. Scores can range from 12 to 84. Higher scores indicate higher procrastination. Example items include; “Even after I make a decision I delay acting upon it.”, and “I often find myself doing tasks that I had intended to do days before.”.

### Statistical analytical approach

In this study, SPSS statistics software was used with PROCESS MACRO [93] to investigate the mediation hypothesis with a bootstrapped multivariate technique. The mediation effects were investigated by testing the indirect effect mindfulness has on procrastination through two mediators; trait anxiety, and maladaptive perfectionism. 95% bias-corrected confidence intervals were calculated with this procedure, and the mediation effect is considered significant if these confidence intervals do not overlap with zero. In all tests, the significance value alpha was fixed at 0.01 to reduce the Type-1 error rate as suggested [94–97] and narrow the decision region using strict criteria.

Before the analyses, normality assumptions were checked. Distribution analyses based on skewness and kurtosis values revealed all individual scales had approximately normal distributions. Both skewness and kurtosis values were between −1.96 and + 1.96, which represents the general threshold for normality assumptions [98]. Normal distribution of data was reinforced by the Q-Q plots. No significant outliers were identified. Therefore, all data were included for further analyses and there were no missing data.

## Results

### Descriptive and correlation analysis

Table 1 demonstrates the descriptive statistics and correlations between self-reported dispositional mindfulness, trait anxiety, maladaptive perfectionism, and procrastination among the participants. Results indicated that (a) higher mindfulness was associated with lower procrastination, lower trait anxiety, and lower maladaptive perfectionism(b) higher procrastination was associated with increased trait anxiety and increased maladaptive perfectionism, and (c) higher trait anxiety was associated with higher maladaptive perfectionism.

**Table 1:** Mean, Standard Deviation, Skewness, Kurtosis, and Pearson Correlation Matrix of The Variables of Interest.

	*M*	*SD*	*Skewness*	*Kurtosis*	FFMQ	STAI-T	APS_ Discrepancy	PPS
FFMQ-15	47.18	6.59	−.14	−.11	1	−.60	−.48	−.39
STAI-T	47.52	10.42	−.22	−.72		1	.69	.54
APS_D	51.67	14.49	−.17	−.44			1	.61
PPS	52.22	14.08	−.22	−.14				1

*Note.* All correlations are significant at *p < *.001*.* N = 126; *M* = Means; *SD*=Standard Deviations. FFMQ-15 = 15-Item Five Facet Mindfulness Questionnaire; STAI-T = State-Trait Anxiety Inventory-Trait; APS_D = Almost Perfect Scale Revised_Discrepancy subscale; PPS=Pure Procrastination Scale.

Separate one-way ANOVA tests were conducted to investigate whether gender, student level, and ethnicity had any significant effect on the study variables. The significant alpha value was set at the.01 level due to conducting multiple comparisons, which is known to increase the risk of Type-1 errors [99,100]. Alongside the assumption of normality, the homogeneity of variances was checked before the ANOVAs. According to the Levene’s test based on mean with 95% CI, variance homogeneity assumptions were met (*p* > .01). The ANOVA results, as demonstrated in Table 2, revealed that there were no significant differences in procrastination, maladaptive perfectionism, trait anxiety or dispositional mindfulness based on gender, student level or ethnicity (*p* > .01). In addition, Pearson Product Moment correlation analysis was conducted to test whether there is a relationship between age, which is a continuously coded variable, and the study variables. No significant correlation was found between age and procrastination (*p* = .367), maladaptive perfectionism (*p* = .115), trait anxiety (*p* = .311), and dispositional mindfulness (*p* = .033). Therefore, no covariates were identified for further analyses.

**Table 2:** Examination of Mean Differences Based on Descriptive Variables.

	Gender		Ethnicity		Student Status	
	*F* ^*a*^	*p*	*F* ^*b*^	*p*	*F* ^*c*^	*p*
PPS	.52	.473	1.16	.334	.48	.698
APS_D	.12	.733	.53	.755	2.69	.050
STAI-T	3.56	.061	.40	.848	.96	.414
FFMQ-15	6.08	.015	.87	.504	2.38	.073

Note PPS=Pure Procrastination Scale; APS_D = Almost Perfect Scale Revised_Discrepancy subscale; STAI-T = State-Trait Anxiety Inventory-Trait; FFMQ-15 = 15-Item Five Facet Mindfulness Questionnaire. *F*^a^ = *F*(1, 124); *F*^b^ = *F*(5, 120); *F*^c^ = *F*(3, 122).

### Mediation analysis

Assumptions for regression were investigated before further analysis. FFMQ-15, APS_Discrepancy and STAI-T were entered into the analysis as independent variables, and PPS as dependent variable. Collinearity diagnostics were run in SPSS to examine whether there was a multicollinearity problem. Accordingly, the tolerance values were between.43 and.64, and the VIF values were between 1.57 and 2.30, indicating that the data met the assumption of collinearity. The correlation of the errors was checked with the Durbin-Watson test, and the data met the assumption of independent errors (Durbin-Watson value =  2.04). In other words, the errors were uncorrelated. An analysis of standard residuals was carried out to check for outliers. The results showed that there were no significant outliers in the data (Std. Residual Min =  −2.85, Std. Residual Max =  2.31). The normal distribution of errors was checked with a histogram and a P-P plot of standardized residuals. Both visuals indicated that the data contained normally distributed errors with no outliers. The scatterplot of standardized residuals and standardized predicted values indicated that the data met the assumption of homoscedasticity of errors and linearity. The data also met the assumption of non-zero variances (FFMQ-15, Variance =  43.46; STAI-T, Variance =  108.49; APS_Discrepancy, Variance =  210.06; PPS, Variance =  198.17). After all the assumptions were met, the mediation model was examined.

Baron and Kenny as cited in Abu-Bader and Jones [101], explain three conditions for mediation. The first two conditions include a significant predictive value of the independent variable on the dependent variable and the mediating variable. The third condition requires the mediator to significantly predict the dependent variable when controlling for the independent variable.

A preliminary regression model was established to examine whether dispositional mindfulness predicted procrastination. A significant effect was observed on the model results as analysis revealed that mindfulness significantly predicted procrastination and explained 15% of the variance (*R*^*2*^ = .15, *F*[1, 124] =  22.16, *p* < .001), please see Table 3.

**Table 3:** Preliminary Regression Analysis Before Mediation Model.

			95%CI				
	Beta	*SE*	*LL*	*UL*	*β*	*t*	*p*
FFMQ-15	−.83	.18	−1.18	−4.8	−.39	−4.71^*^	<.001

*Note.* FFMQ-15=15-Item Five Facet Mindfulness Questionnaire; *SE=*Standard Error; CI = Confidence Interval; *LL* = Lower Limit; *UL* = Upper Limit.

To examine the effects of anxiety and maladaptive perfectionism between dispositional mindfulness and procrastination, a parallel mediation model was tested using bootstrapping technique with PROCESS MACRO in SPSS. With this method, the indirect effects of anxiety and maladaptive perfectionism were estimated separately in five thousand random samples taken from the data. Dispositional mindfulness was the predictor in this model, with trait anxiety and maladaptive perfectionism as the mediators, and procrastination as the outcome variable. Bias-corrected percentile 95% confidence intervals were calculated for the model.

When the overall model summary was examined, it was determined that 40% of the variance in procrastination was significantly explained by the predictors (dispositional mindfulness, trait anxiety and maladaptive perfectionism) (*R*^2^ = .40, *F*[3, 122] =  27.50*, p < *.001*).*

Results of the model indicate that the direct effects of dispositional mindfulness on trait anxiety (*R*^2^ = .35, *F*[1, 124] =  68.32, *p* < .001); maladaptive and perfectionism (*R*^2^ = .23, *F*[1, 124] =  36.73, *p* < .001) are both statistically significant. The model further revealed that the direct effect of dispositional mindfulness on procrastination is no longer significant (*B* =  −.11, *β* =  −.05, *t* =  −.61, *p* = .545, *95% CI* =  [−.48,.26]) when controlled for *t*he mediators (trait anxiety and maladaptive perfectionism). The total indirect effect of dispositional mindfulness on procrastination through the mediators is significant with *β* value at −.34 (*B* =  −.72), and 95% CI is [−1.01, −.44]. The parallel mediation model further revealed that maladaptive perfectionism had a significant mediating effect on the relationship between dispositional mindfulness and procrastination when controlled for trait anxiety. On the other hand, trait anxiety did not significantly mediate the relationship between dispositional mindfulness and procrastination when controlled for maladaptive perfectionism. The results of the parallel mediation model are shown in Table 4.

**Table 4:** Statistical Coefficients of the Parallel Mediation Model.

					95% CI				
*Direct Effects*	*B*	*SE*	*t*	*p* ^*c*^	*LL*	*UL*	*β*	*R* ^*2*^	*p* ^*m*^
FFMQ → STAI-T	−.94	.11	−8.27	<.001	−1.17	−.72	−.60	.35	<.001
FFMQ → APS_D	−1.05	.17	−6.06	<.001	−1.39	−.71	−.48	.23	<.001
FFMQ → PPS	−.11	.19	−.61	.545	−.48	.26	−.05	.40	<.001
STAI-T → PPS	.29	.14	2.01	.047	.00	.57	.21		
APS_D → PPS	.42	.09	4.51	<.001	.24	.61	.44		
Total Effect	*B*	*SE*	*t*	*p* ^*c*^	*LL*	*UL*	*β*	*R* ^*2*^	*p* ^*m*^
FFMQ → PPS	−.83	.18	−4.71	<.001	−1.18	−.48	−.39	.15	<.001
		95% CI							
*Indirect Effects*	*B*	*LL*	*UL*	*β*					
TOTAL	−.72	−1.01	−.44	−.34					
FFMQ → STAI-T → PPS	−.27	−.55	.02	−.13					
FFMQ → APS_D → PPS	−.45	−.69	−.24	−.21					

*Note*. FFMQ = 15-Item Five-Facet Mindfulness Questionnaire; STAIT-T = State-Trait Anxiety Inventory-Trait; APS_D = Almost Perfect Scale Revised_Discrepancy subscale; PPS=Pure Procrastination Scale. *SE=*Standard Error. *p* ^*c*^ =  p value of coefficients; *p* ^*m*^ =  p value of the model summary; CI =  Confidence Interval; *LL* =  Lower Limit; *UL* =  Upper Limit.

**Fig 2:**
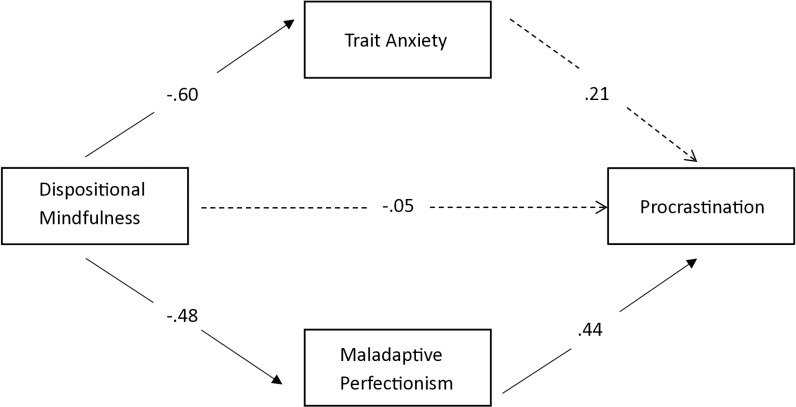
Parallel Mediation Diagram Showing Standard Regression Coefficients. The dashed lines indicate nonsignificant coefficients.

Thus, from the parallel mediation analysis using the bootstrap confidence intervals, it is concluded that maladaptive perfectionism fully mediates the relationship between dispositional mindfulness and procrastination. Please see Fig 2 for the model summary.

## General discussion

The current study investigated the mediating role of maladaptive perfectionism and trait anxiety to explain the relationship between dispositional mindfulness and procrastination by testing a parallel mediation model with a bootstrapped multivariate technique. In line with the previous research and theory, it was hypothesized that the inverse relationship between dispositional mindfulness and procrastination could be explained by the mediating roles of maladaptive perfectionism and trait anxiety. The results of the model partially support our hypothesis and highlight that dispositional mindfulness links to procrastination primarily through the mediator of maladaptive perfectionism as the mediating effect of trait anxiety is non-significant when controlled for maladaptive perfectionism. The findings reveal that maladaptive perfectionism fully mediates the relationship between dispositional mindfulness and procrastination. In other words, increased levels of dispositional mindfulness only indirectly link to decreased levels of procrastination through subsequent reductions in maladaptive perfectionism.

### Mindfulness and procrastination

As expected, preliminary regression analysis revealed that dispositional mindfulness significantly and inversely predicted procrastination. This finding is not surprising considering Kabat-Zinn’s work as cited in Shapiro and Schwartz [29] proposes mindfulness to be an efficient self-regulation technique through its explicit focus on acceptance, present moment awareness, and directing conscious attention to thoughts and emotions. It is argued that people who procrastinate tend to avoid unpleasant emotions or thoughts [15], and it is logical to assume mindfulness which fosters acceptance and paying attention to the present moment in a nonjudgemental manner would be linked to less procrastination. As such, this is consistent with the research evidence suggesting that higher levels of dispositional mindfulness are associated with less procrastination [14,15,33–36]. Building on this inverse relationship between dispositional mindfulness and procrastination, and research showing that mindfulness interventions effectively decrease procrastination [38,39], it has been argued that mindfulness could have a unique influence on procrastination [34].

The findings of the current study contradict the suggestion that mindfulness can be a unique and significant predictor of procrastination. It is important to highlight that these findings partially contradict with the study by Gautam and colleagues [40]. This study is important to mention because, to the best of the authors’ knowledge, it is the only study investigating a potential mediator, *anxiety*, to explain the relationship between dispositional mindfulness and procrastination. Gautam and colleagues [40], showed that all facets of mindfulness except observing had a significant indirect effect on procrastination through anxiety. However, the authors have also indicated that there were significant direct effects of some facets of dispositional mindfulness (acting with awareness and observing) on procrastination. It was also demonstrated that dispositional mindfulness uniquely and significantly added to the prediction of procrastination. However, this contribution was relatively small and explained an additional 0.4% of the variance in procrastination. It is also important to highlight that even though this study was conducted on a large sample and included facets of mindfulness in statistical analysis rather than an overall mindfulness score, the generalizability of the results is restricted to students and academic procrastination only. Another important point to be made is that unlike Gautam and colleagues [40], the current study has also included an additional measurement of maladaptive perfectionism. We believe it is the critical addition of maladaptive perfectionism that explains our lack of convergences with the results of Gautam and colleagues [40].

### The mediating role of trait anxiety and maladaptive perfectionism

From the perspective of the previously mentioned Self-Regulation Theory, procrastination can be explained as a dysfunctional response to negative affect [25]. The findings of the current study highlight that while trait anxiety is a part of the model and possibly could be an important variable in explaining the variance in procrastination, its mediating role is not statistically significant when controlled for maladaptive perfectionism. At an individual level, the findings of the parallel mediation model indicated that trait anxiety did not significantly predict procrastination (*β* = .21, *B* = .29, *t* =  2.01, *p* = .047, *95% CI = * [.00, .57]). It is important to note that the current study strictly adopted the significance level at.01 in the analyses which led the authors to conclude that the effect of trait anxiety on procrastination was nonsignificant with the p-value at.05. On the other hand, Pearson correlation reinforced a positive and significant relationship between the two variables (*r* = .54, *p* < .001) This result is consistent with the previous research supporting that higher levels of trait anxiety are associated with more procrastination [4,35,42–45]. As suggested by several authors [2,27], it seems possible that people tend to disengage with the task and procrastinate following negative affect (i.e., anxiety). Tice and Bratslavsky as cited in Eckert and colleagues [25], suggested that individuals engage in procrastination to achieve short-term mood repair. In other words, it might be the case that people procrastinate following negative affect because they choose a temporary relief from distress, and therefore avoid the task that brings this psychological distress [102]. However, it is important to note that the current study does not support the causal theoretical claims and highlights that the relationship between trait anxiety and procrastination is solely correlational. It is also important to emphasize that the current study particularly focuses on trait anxiety, described as an individual’s general tendency to be anxious, and does not speculate on any other types of anxiety.

It was hypothesized that dispositional mindfulness links to procrastination via altered levels of trait anxiety and maladaptive perfectionism. Numerous meta-analyses previously supported that increased levels of dispositional mindfulness lead to lower levels of trait anxiety [49–51] which can be explained by improved emotional regulation [52,54]. Results of the current study support this relationship between dispositional mindfulness and trait anxiety, highlighting that dispositional mindfulness significantly and negatively predicts trait anxiety (*R*^2^ = .35, *β* =  −.60, *95% CI* =  [−1.17, −.72], *p* < .001). Overall, while anxiety is part of the model, its mediating role is not statistically significant when controlled for maladaptive perfectionism.

When it comes to maladaptive perfectionism, the results highlight the mediating role of maladaptive perfectionism is significant and accounts for a substantial portion of the total effect in explaining the mindfulness-procrastination link. It was revealed that dispositional mindfulness links to procrastination primarily through the mediator of maladaptive perfectionism and this variable fully mediated the relationship between dispositional mindfulness and procrastination.

In the current study, the operational definition provided by Slaney and colleagues [57] for maladaptive perfectionism defined as *perceived discrepancies between an individual’s standards and their actual performance* was followed*.* The APS discrepancy subscale used in this study included both emotional (i.e., “I often feel frustrated because I can’t meet my goals.”) and cognitive components (i.e., “Doing my best never seems to be enough.”) of maladaptive perfectionism. As expected, the findings revealed that maladaptive perfectionism significantly and positively predicts procrastination with *β* value at*.44 (B = .42, p* < .001*) and* 95% CI of the direct effect is [.24, .61]. This finding is in line with the previously mentioned theory of Self-Regulation, specifically Carver and Scheier’s Cybernetic Self-Regulation Theory as cited in Carver [26]. Within this model, the relevant *expectancy-assessment process* highlighted the importance of believing that the difference between one’s current state and the desired state can be reduced. This process seems to be especially important in linking perfectionism and procrastination. Considering that perfectionism involves setting unrealistically high standards, and maladaptive perfectionism involves the perceived discrepancies between these high standards and the actual state, it is believed there is a greater expectancy of not being able to reduce the discrepancies which leads to disengagement, and subsequently affects procrastination. However, these ideas should be approached with caution. Even though the Cybernetic Self-Regulation Theory proposes that perfectionism and procrastination can be linked through this expectancy-assessment process, this process was not assessed in the current study, and thus although a potential explanation it remains speculation. As such, Slade and Owens as cited in Shih [75] provide another possible explanation for the relationship between perfectionism and procrastination. They propose that adaptive perfectionism is marked by the motivation to approach success, whereas maladaptive perfectionism is likely to be linked with the motivation to avoid failure. In turn, it is suggested that this avoidance motivation may exaggerate procrastination [75].

Overall, the finding supporting a positive link between maladaptive perfectionism and procrastination is in line with previous research [61,62,64,67,72–76,78]. On the other hand, it is important to mention Steel’s [2] meta-analysis which concluded that there was no significant association between perfectionism and procrastination. However, this meta-analysis had a potential methodological problem because in that it did not differentiate between adaptive and maladaptive perfectionism. It is argued that by combining the two dimensions of perfectionism into one variable or not being conscious of the multidimensionality of perfectionism, Steel potentially confounded the relationship between perfectionism and procrastination [73].

It is important to mention that there are substantial differences between previously presented literature and the current study. To the best of our knowledge, all studies including measurements of perfectionism and procrastination were conducted on student samples [61,62,64,67,72,74–76,78]. Not surprisingly, an important part of these studies has specifically focused on academic procrastination [61,64,75,78]. Moreover, most of the studies were not consistent when it comes to how they described maladaptive perfectionism. Two studies [61,64] have adopted the same definition the current study has, and assessed maladaptive perfectionism as *discrepancies* with the APS discrepancy subscale. Others [62,67,72–74,76,78] have adopted different definitions for maladaptive perfectionism. For example; both Mushquash and Sherry [67] and Closson and Boutilier [62] referred to maladaptive perfectionism as socially-prescribed perfectionism*.* However, according to Frost and colleagues as cited in Sederlund and colleagues [58], even though both discrepancies and socially prescribed perfectionism reflect the maladaptive aspect of perfectionism, and both are referred to as maladaptive perfectionism, the two constructs are substantially different from each other. According to Hewitt and Flett as cited in Slaney and colleagues [57], socially prescribed perfectionism is *believing that other people have extremely high standards for an individual to be perfect* whereas discrepancies do not make any assumptions regarding individual beliefs of other people’s expectations. The APS discrepancy subscale used in the current study only measures the perceived discrepancies for the individual with items such as “I rarely live up to my high standards.”, and does not assess beliefs about other people’s expectations.

Overall, research supporting the positive link between maladaptive perfectionism and procrastination has adopted different measurements and different definitions. Subsequently, even though there are overlaps among the different definitions of maladaptive perfectionism, the evidence is restricted to how maladaptive perfectionism is defined in individual studies and we believe that it should not generalize to others that have adopted a different operational definition. Subsequently, the current literature only provides evidence from a handful of studies to support the relationship between discrepancies specifically and procrastination. However, the current research expands the evidence for the discrepancies-procrastination link alongside others [61,64]

Despite the differences in methodology, the finding highlighting that maladaptive perfectionism positively predicts procrastination is consistent with the evidence identifying maladaptive perfectionism as an important factor linked to procrastination. Moreover, the current study goes beyond the current literature by investigating the link between maladaptive perfectionism and procrastination in an adult sample not limited to students and further focuses on everyday procrastination.

## Limitations, and strengths

It is important to acknowledge certain limitations of the present study. First of all, the current study relied on self-report questionnaires which always pose a risk of invalid or untruthful answers [103]. However, we believe that ensuring confidentiality and anonymity would be effective in preventing social-desirability bias. Secondly, due to the cross-sectional design of the study, it is not possible to make any causal interpretations. Even though this research speculates that dispositional mindfulness links to procrastination via reduced levels of maladaptive perfectionism based on theoretical accounts and research evidence, longitudinal and experimental designs are necessary to support this. We hope that this work provides a direction for future longitudinal and experimental studies to draw causal explanations regarding the variables included in this study. Based on the evidence and theoretical background provided in addition to the mediation analyses conducted, we believe that mindfulness still has the potential to decrease procrastination via its negative associations with maladaptive perfectionism. At this point, the findings add to the current literature but should be treated with caution. We suggest that future research should investigate the influence of mindfulness on procrastination via mindfulness-based interventions, and include measurements of trait anxiety and maladaptive perfectionism in experimental or longitudinal research designs to determine their effects when controlled for one another. We suggest future studies be mindful of the significant and strong correlation observed between trait anxiety and maladaptive perfectionism (*r* = .69, *p* < .001) and account for both variables in their models. We believe including only one of these variables in models aiming to predict procrastination or the relationship between dispositional mindfulness and procrastination could likely taint the accuracy of the findings and provide fragmentary explanations.

When it comes to the external validity of the results, the age of the participants ranged from 18 to 33. Therefore, these results cannot be generalized to other age populations. Similarly, the particular sample was mostly comprised of females (*N* =  83). With regard to gender, the data did not reveal any group differences in any of the study variables. Regardless, generalization across genders should be approached with caution. Moreover, the generalization of the results is limited to a small sample size, and replication of the results is needed with larger populations. Additionally, more than half of the participants were undergraduate students, almost a quarter were not students, and the rest were postgraduate students. However, no significant group differences were found in any of the study variables. Accordingly, we believe that these findings can be generalized to this particular age group regardless of student status or gender if further research can support that there are no group differences between undergraduate students, postgraduate students, and non-students. Nevertheless, more research is needed to determine if these results can be replicated in different populations. Finally, on that note, this study considered dispositional mindfulness in the form of an overall score, we suggest that future research could further investigate how different facets of mindfulness relate to trait anxiety and maladaptive perfectionism, and in turn procrastination. Particularly given, Gautam and colleagues [40], showed that some facets of dispositional mindfulness might not reveal significant relationships with anxiety, or procrastination. The effects of particular facets of mindfulness could be more profound in relation to these variables. Investigating the effects of each facet could give further insight into the relationships among the study variables. That said, we believe this could provide invaluable insight when it comes to developing mindfulness-based interventions to improve maladaptive perfectionism, and thus procrastination.

## Conclusion

Given the widespread occurrence of procrastination and its negative associations ranging from psychological distress [10], to poorer physical health [15], it is important to understand the variables that contribute to procrastination. The current study highlights that the general tendency to be mindful in daily life (dispositional mindfulness) [29,85] can negatively predict procrastination. To put it simply, people who are more mindful in daily life procrastinate less. However, as the literature suggests, it is unclear whether this can be attributed to dispositional mindfulness itself or other factors that might be in the middle of this relationship. The current study indeed shows that the relationship between dispositional mindfulness and procrastination can be explained by an important mechanism: the perceived difference between one’s standards and one’s actual performance (maladaptive perfectionism). The results of the study show that higher daily mindfulness only indirectly links to less procrastination through decreased levels of one’s perceived discrepancies between their standards and their actual performance. In other words, improved dispositional mindfulness only links to less procrastination if it is associated with decreased levels of maladaptive perfectionism. Despite these important findings, there is still much to learn about procrastination which also highlights the importance of investigating the possibility of other explanatory variables.

To the best of our knowledge, this study is the first to investigate maladaptive perfectionism as a possible mechanism to explain the relationship between dispositional mindfulness and procrastination. As our study highlights the importance of maladaptive perfectionism to explain the link between mindfulness and procrastination, we suggest that this important variable be included in the theoretical models aimed at predicting procrastination and future studies investigate the predictive values of both maladaptive perfectionism and trait anxiety in explaining the variance in procrastination individually and comparably.

## Supporting information

S1 File Codebook for Data Set. (DOCX)

S2 File Data Set. (SAV)
