# Correction: Maladaptive perfectionism can explain the inverse relationship between dispositional mindfulness and procrastination

**DOI:** 10.1371/journal.pone.0322030

**Published:** 2025-04-04

**Authors:** Gozde Ibili, Jeremy John Tree, Yılmaz Orhun Gurluk

The ORCID iD is missing for the third author. Please see the third author’s ORCID iD here:

- Author Yılmaz Orhun Gurluk’s ORCID iD is: 0000-0002-1134-3776 (https://orcid.org/0000-0002-1134-3776).
