# Supplementary material for: Maladaptive perfectionism can explain the inverse relationship between dispositional mindfulness and procrastination
Source: PLoS One. 2025 Feb 12;20(2):e0318845. doi: 10.1371/journal.pone.0318845 (PMC11819553; doi:10.1371/journal.pone.0318845)
Supplement: S1 File — (DOCX) [file pone.0318845.s001.docx]

| **Variable Name** | **Position** | **Type** | **Label** | **Values** | **Missing** | **Measurement Level** | **Role** |
| --- | --- | --- | --- | --- | --- | --- | --- |
| StartDate | 1 | Numeric | When the survey was started. | None | None | Scale | Input |
| End Date | 2 | Numeric | When the survey was completed. | None | None | Scale | Input |
| Progress | 3 | Numeric | How much percentage of the survey was completed. | None | None | Scale | Input |
| Duration__in_seconds_ | 4 | Numeric | How many seconds it took to complete the survey. | None | None | Scale | Input |
| Finished | 5 | Numeric | The survey was completed. | 0: False  1: True | None | Scale | Input |
| RecordedDate | 6 | Numeric | When the survey was recorded. | None | None | Scale | Input |
| LocationLatitude | 7 | String | Location latitude of the participants at the time of survey completion. | None | None | Nominal | Input |
| LocationLongitude | 8 | String | Location longitude of the participants at the time of survey completion. | None | None | Nominal | Input |
| DistributionChannel | 9 | String | Distribution channel. | None | None | Nominal | Input |
| UserLanguage | 10 | String | The language the survey was completed in. | None | None | Nominal | Input |
| acceptance | 11 | Numeric | Acceptance of the participant consent form (S2_file) | 1: Yes, I agree with all statements listed above.  2: No, I disagree with any of the statements above. | None | Scale | Input |
| age | 12 | Numeric | What is your age (in numbers)? | None | None | Scale | Input |
| gender | 13 | Numeric | What is your gender (as in birth)? | 1: Male  2: Female  3: Other (please specify)  4: Prefer not to say | None | Scale | Input |
| ethnicity | 14 | Numeric | What is your ethnicity? | 1: White  2: Black or African American  3: American Indian or Alaska Native  4: Asian  5: Native Hawaiian or Pacific Islander  6: Hispanic or Latino  7: Other (please specify)  8: Prefer not to say | None | Scale | Input |
| student_status | 15 | Numeric | If you are currently a student at the university, please state your student status (if you are not a student at the university, skip the question). | 1: Undergraduate Student  2: Postgraduate Student  3: Other (please specify)  4: Not student | None | Scale | Input |
| Q6_1 | 16 | Numeric | Please use the scale provided to indicate how true the below statement are of you. Select the option which represents your own opinion of what is generally true for you. -When I take a shower or a bath, I stay alert to the sensations of water on my body. | 1: Never or very rarely true  2: Rarely true  3: Sometimes true  4: Often true  5: Very often or always true | None | Scale | Input |
| Q6_2 | 17 | Numeric | Please use the scale provided to indicate how true the below statement are of you. Select the option which represents your own opinion of what is generally true for you. – I’m good at finding words to describe my feelings. | 1: Never or very rarely true  2: Rarely true  3: Sometimes true  4: Often true  5: Very often or always true | None | Scale | Input |
| Q6_3 | 18 | Numeric | Please use the scale provided to indicate how true the below statement are of you. Select the option which represents your own opinion of what is generally true for you. – I don’t pay attention to what I’m doing because I’m daydreaming, worrying, or otherwise distracted. | 1: Very often or always true  2: Often true  3: Sometimes true  4: Rarely true  5: Never or very rarely true | None | Scale | Input |
| Q6_4 | 19 | Numeric | Please use the scale provided to indicate how true the below statement are of you. Select the option which represents your own opinion of what is generally true for you. -I believe some of my thoughts are abnormal or bad and I shouldn’t think that way. | 1: Very often or always true  2: Often true  3: Sometimes true  4: Rarely true  5: Never or very rarely true | None | Scale | Input |
| Q6_5 | 20 | Numeric | Please use the scale provided to indicate how true the below statement are of you. Select the option which represents your own opinion of what is generally true for you. – When I have distressing thoughts or images, I “step back” and am aware of the thought or image without getting taken over by it. | 1: Never or very rarely true  2: Rarely true  3: Sometimes true  4: Often true  5: Very often or always true | None | Scale | Input |
| Q6_6 | 21 | Numeric | Please use the scale provided to indicate how true the below statement are of you. Select the option which represents your own opinion of what is generally true for you. – I notice how foods and drinks affect my thoughts, bodily sensations, and emotions. | 1: Never or very rarely true  2: Rarely true  3: Sometimes true  4: Often true  5: Very often or always true | None | Scale | Input |
| Q6_7 | 22 | Numeric | Please use the scale provided to indicate how true the below statement are of you. Select the option which represents your own opinion of what is generally true for you. – I have trouble thinking of the right words to express how I feel about things. | 1: Very often or always true  2: Often true  3: Sometimes true  4: Rarely true  5: Never or very rarely true | None | Scale | Input |
| Q6_8 | 23 | Numeric | Please use the scale provided to indicate how true the below statement are of you. Select the option which represents your own opinion of what is generally true for you. – I do jons or tasks automatically without being aware of what I’m doing. | 1: Very often or always true  2: Often true  3: Sometimes true  4: Rarely true  5: Never or very rarely true | None | Scale | Input |
| Q6_9 | 24 | Numeric | Please use the scale provided to indicate how true the below statement are of you. Select the option which represents your own opinion of what is generally true for you. – I think some of my emotions are bad or inappropriate and I shouldn’t feel them. | 1: Very often or always true  2: Often true  3: Sometimes true  4: Rarely true  5: Never or very rarely true | None | Scale | Input |
| Q6_10 | 25 | Numeric | Please use the scale provided to indicate how true the below statement are of you. Select the option which represents your own opinion of what is generally true for you. – When I have distressing thoughts or images I am able just to notice them without reacting. | 1: Never or very rarely true  2: Rarely true  3: Sometimes true  4: Often true  5: Very often or always true | None | Scale | Input |
| Q6_11 | 26 | Numeric | Please use the scale provided to indicate how true the below statement are of you. Select the option which represents your own opinion of what is generally true for you. – I pay attention to sensations, such as the wind in my hair or sun on my face. | 1: Never or very rarely true  2: Rarely true  3: Sometimes true  4: Often true  5: Very often or always true | None | Scale | Input |
| Q6_12 | 27 | Numeric | Please use the scale provided to indicate how true the below statement are of you. Select the option which represents your own opinion of what is generally true for you. – Even when I’m feeling terribly upset I can find a way to put it into words. | 1: Never or very rarely true  2: Rarely true  3: Sometimes true  4: Often true  5: Very often or always true | None | Scale | Input |
| Q6_13 | 28 | Numeric | Please use the scale provided to indicate how true the below statement are of you. Select the option which represents your own opinion of what is generally true for you. – I find myself doing things without paying attention. | 1: Very often or always true  2: Often true  3: Sometimes true  4: Rarely true  5: Never or very rarely true | None | Scale | Input |
| Q6_14 | 29 | Numeric | Please use the scale provided to indicate how true the below statement are of you. Select the option which represents your own opinion of what is generally true for you. – I tell myself I shouldn’t be feeling the way I’m feeling. | 1: Very often or always true  2: Often true  3: Sometimes true  4: Rarely true  5: Never or very rarely true | None | Scale | Input |
| Q6_15 | 30 | Numeric | Please use the scale provided to indicate how true the below statement are of you. Select the option which represents your own opinion of what is generally true for you. – When I have distressing thoughts or images I just notice them and let them go. | 1: Never or very rarely true  2: Rarely true  3: Sometimes true  4: Often true  5: Very often or always true | None | Scale | Input |
| Q7_1 | 31 | Numeric | Please read each below statement and use the scale to indicate how you generally feel. There are no right or wrong answers. Do not spend too much time on any statement but give the answer which seems to describe how you generally feel. – I feel pleasant | 1: Almost always  2: Often  3: Sometimes  4: Almost never | None | Scale | Input |
| Q7_2 | 32 | Numeric | Please read each below statement and use the scale to indicate how you generally feel. There are no right or wrong answers. Do not spend too much time on any statement but give the answer which seems to describe how you generally feel. – I feel nervous and restless | 1: Almost never  2: Sometimes  3: Often  4: Almost always | None | Scale | Input |
| Q7_3 | 33 | Numeric | Please read each below statement and use the scale to indicate how you generally feel. There are no right or wrong answers. Do not spend too much time on any statement but give the answer which seems to describe how you generally feel. – I feel satisfied with myself | 1: Almost always  2: Often  3: Sometimes  4: Almost never | None | Scale | Input |
| Q7_4 | 34 | Numeric | Please read each below statement and use the scale to indicate how you generally feel. There are no right or wrong answers. Do not spend too much time on any statement but give the answer which seems to describe how you generally feel. – I wish I could be as happy as others seem to be | 1: Almost never  2: Sometimes  3: Often  4: Almost always | None | Scale | Input |
| Q7_5 | 35 | Numeric | Please read each below statement and use the scale to indicate how you generally feel. There are no right or wrong answers. Do not spend too much time on any statement but give the answer which seems to describe how you generally feel. – I feel like a failure | 1: Almost never  2: Sometimes  3: Often  4: Almost always | None | Scale | Input |
| Q7_6 | 36 | Numeric | Please read each below statement and use the scale to indicate how you generally feel. There are no right or wrong answers. Do not spend too much time on any statement but give the answer which seems to describe how you generally feel. – I feel rested | 1: Almost always  2: Often  3: Sometimes  4: Almost never | None | Scale | Input |
| Q7_7 | 37 | Numeric | Please read each below statement and use the scale to indicate how you generally feel. There are no right or wrong answers. Do not spend too much time on any statement but give the answer which seems to describe how you generally feel. – I am “calm, cool and collected” | 1: Almost always  2: Often  3: Sometimes  4: Almost never | None | Scale | Input |
| Q7_8 | 38 | Numeric | Please read each below statement and use the scale to indicate how you generally feel. There are no right or wrong answers. Do not spend too much time on any statement but give the answer which seems to describe how you generally feel. – I feel that difficulties are piling up so that I cannot overcome them | 1: Almost never  2: Sometimes  3: Often  4: Almost always | None | Scale | Input |
| Q7_9 | 39 | Numeric | Please read each below statement and use the scale to indicate how you generally feel. There are no right or wrong answers. Do not spend too much time on any statement but give the answer which seems to describe how you generally feel. – I worry too much over something that really doesn’t matter | 1: Almost never  2: Sometimes  3: Often  4: Almost always | None | Scale | Input |
| Q7_10 | 40 | Numeric | Please read each below statement and use the scale to indicate how you generally feel. There are no right or wrong answers. Do not spend too much time on any statement but give the answer which seems to describe how you generally feel. – I am happy | 1: Almost always  2: Often  3: Sometimes  4: Almost never | None | Scale | Input |
| Q7_11 | 41 | Numeric | Please read each below statement and use the scale to indicate how you generally feel. There are no right or wrong answers. Do not spend too much time on any statement but give the answer which seems to describe how you generally feel. – I have disturbing thoughts | 1: Almost never  2: Sometimes  3: Often  4: Almost always | None | Scale | Input |
| Q7_12 | 42 | Numeric | Please read each below statement and use the scale to indicate how you generally feel. There are no right or wrong answers. Do not spend too much time on any statement but give the answer which seems to describe how you generally feel. – I lack self-confidence | 1: Almost never  2: Sometimes  3: Often  4: Almost always | None | Scale | Input |
| Q7_13 | 43 | Numeric | Please read each below statement and use the scale to indicate how you generally feel. There are no right or wrong answers. Do not spend too much time on any statement but give the answer which seems to describe how you generally feel. – I feel secure | 1: Almost always  2: Often  3: Sometimes  4: Almost never | None | Scale | Input |
| Q7_14 | 44 | Numeric | Please read each below statement and use the scale to indicate how you generally feel. There are no right or wrong answers. Do not spend too much time on any statement but give the answer which seems to describe how you generally feel. – I make decisions easily | 1: Almost always  2: Often  3: Sometimes  4: Almost never | None | Scale | Input |
| Q7_15 | 45 | Numeric | Please read each below statement and use the scale to indicate how you generally feel. There are no right or wrong answers. Do not spend too much time on any statement but give the answer which seems to describe how you generally feel. – I feel inadequate | 1: Almost never  2: Sometimes  3: Often  4: Almost always | None | Scale | Input |
| Q7_16 | 46 | Numeric | Please read each below statement and use the scale to indicate how you generally feel. There are no right or wrong answers. Do not spend too much time on any statement but give the answer which seems to describe how you generally feel. – I am content | 1: Almost always  2: Often  3: Sometimes  4: Almost never | None | Scale | Input |
| Q7_17 | 47 | Numeric | Please read each below statement and use the scale to indicate how you generally feel. There are no right or wrong answers. Do not spend too much time on any statement but give the answer which seems to describe how you generally feel. – Some unimportant thought runs through my mind and bothers me | 1: Almost never  2: Sometimes  3: Often  4: Almost always | None | Scale | Input |
| Q7_18 | 48 | Numeric | Please read each below statement and use the scale to indicate how you generally feel. There are no right or wrong answers. Do not spend too much time on any statement but give the answer which seems to describe how you generally feel. – I take disappointments so keenly that I can’t put them out of my mind | 1: Almost never  2: Sometimes  3: Often  4: Almost always | None | Scale | Input |
| Q7_19 | 49 | Numeric | Please read each below statement and use the scale to indicate how you generally feel. There are no right or wrong answers. Do not spend too much time on any statement but give the answer which seems to describe how you generally feel. – I am a steady person | 1: Almost always  2: Often  3: Sometimes  4: Almost never | None | Scale | Input |
| Q7_20 | 50 | Numeric | Please read each below statement and use the scale to indicate how you generally feel. There are no right or wrong answers. Do not spend too much time on any statement but give the answer which seems to describe how you generally feel. – I get in a state of tension or turmoil as I think over my recent concerns and interests | 1: Almost never  2: Sometimes  3: Often  4: Almost always | None | Scale | Input |
| Q8_1 | 51 | Numeric | The following items are designed to measure attitudes people have towards themselves, their performance, and toward others. There are no right or wrong answers. Please respons to all of the items. Use your first impression and do not spend too much time on individual items in responding. Respond to each of the items using the scale below to describe your degree of agreement with each item. – I have high standards for my performance at work or at school. | 1: Strongly disagree  2: Disagree  3: Slightly disagree  4: Neutral  5: Slightly agree  6: Agree  7: Strongly agree | None | Scale | Input |
| Q8_2 | 52 | Numeric | The following items are designed to measure attitudes people have towards themselves, their performance, and toward others. There are no right or wrong answers. Please respons to all of the items. Use your first impression and do not spend too much time on individual items in responding. Respond to each of the items using the scale below to describe your degree of agreement with each item. – I am an orderly person. | 1: Strongly disagree  2: Disagree  3: Slightly disagree  4: Neutral  5: Slightly agree  6: Agree  7: Strongly agree | None | Scale | Input |
| Q8_3 | 53 | Numeric | The following items are designed to measure attitudes people have towards themselves, their performance, and toward others. There are no right or wrong answers. Please respons to all of the items. Use your first impression and do not spend too much time on individual items in responding. Respond to each of the items using the scale below to describe your degree of agreement with each item. – I often feel frustrated because I can’t meet my goals. | 1: Strongly disagree  2: Disagree  3: Slightly disagree  4: Neutral  5: Slightly agree  6: Agree  7: Strongly agree | None | Scale | Input |
| Q8_4 | 54 | Numeric | The following items are designed to measure attitudes people have towards themselves, their performance, and toward others. There are no right or wrong answers. Please respons to all of the items. Use your first impression and do not spend too much time on individual items in responding. Respond to each of the items using the scale below to describe your degree of agreement with each item. – Neatness is important to me. | 1: Strongly disagree  2: Disagree  3: Slightly disagree  4: Neutral  5: Slightly agree  6: Agree  7: Strongly agree | None | Scale | Input |
| Q8_5 | 55 | Numeric | The following items are designed to measure attitudes people have towards themselves, their performance, and toward others. There are no right or wrong answers. Please respons to all of the items. Use your first impression and do not spend too much time on individual items in responding. Respond to each of the items using the scale below to describe your degree of agreement with each item. – If you don’t expect much out of yourself, you will never succeed. | 1: Strongly disagree  2: Disagree  3: Slightly disagree  4: Neutral  5: Slightly agree  6: Agree  7: Strongly agree | None | Scale | Input |
| Q8_6 | 56 | Numeric | The following items are designed to measure attitudes people have towards themselves, their performance, and toward others. There are no right or wrong answers. Please respons to all of the items. Use your first impression and do not spend too much time on individual items in responding. Respond to each of the items using the scale below to describe your degree of agreement with each item. – My best just never seems to be good enough for me. | 1: Strongly disagree  2: Disagree  3: Slightly disagree  4: Neutral  5: Slightly agree  6: Agree  7: Strongly agree | None | Scale | Input |
| Q8_7 | 57 | Numeric | The following items are designed to measure attitudes people have towards themselves, their performance, and toward others. There are no right or wrong answers. Please respons to all of the items. Use your first impression and do not spend too much time on individual items in responding. Respond to each of the items using the scale below to describe your degree of agreement with each item. – I think things should be put away in their place. | 1: Strongly disagree  2: Disagree  3: Slightly disagree  4: Neutral  5: Slightly agree  6: Agree  7: Strongly agree | None | Scale | Input |
| Q8_8 | 58 | Numeric | The following items are designed to measure attitudes people have towards themselves, their performance, and toward others. There are no right or wrong answers. Please respons to all of the items. Use your first impression and do not spend too much time on individual items in responding. Respond to each of the items using the scale below to describe your degree of agreement with each item. – I have high expectations for myself. | 1: Strongly disagree  2: Disagree  3: Slightly disagree  4: Neutral  5: Slightly agree  6: Agree  7: Strongly agree | None | Scale | Input |
| Q8_9 | 59 | Numeric | The following items are designed to measure attitudes people have towards themselves, their performance, and toward others. There are no right or wrong answers. Please respons to all of the items. Use your first impression and do not spend too much time on individual items in responding. Respond to each of the items using the scale below to describe your degree of agreement with each item. – I rarely live up to my high standards. | 1: Strongly disagree  2: Disagree  3: Slightly disagree  4: Neutral  5: Slightly agree  6: Agree  7: Strongly agree | None | Scale | Input |
| Q8_10 | 60 | Numeric | The following items are designed to measure attitudes people have towards themselves, their performance, and toward others. There are no right or wrong answers. Please respons to all of the items. Use your first impression and do not spend too much time on individual items in responding. Respond to each of the items using the scale below to describe your degree of agreement with each item. – I like to always be organized and disciplined. | 1: Strongly disagree  2: Disagree  3: Slightly disagree  4: Neutral  5: Slightly agree  6: Agree  7: Strongly agree | None | Scale | Input |
| Q8_11 | 61 | Numeric | The following items are designed to measure attitudes people have towards themselves, their performance, and toward others. There are no right or wrong answers. Please respons to all of the items. Use your first impression and do not spend too much time on individual items in responding. Respond to each of the items using the scale below to describe your degree of agreement with each item. – Doing my best never seems to be enough. | 1: Strongly disagree  2: Disagree  3: Slightly disagree  4: Neutral  5: Slightly agree  6: Agree  7: Strongly agree | None | Scale | Input |
| Q8_12 | 62 | Numeric | The following items are designed to measure attitudes people have towards themselves, their performance, and toward others. There are no right or wrong answers. Please respons to all of the items. Use your first impression and do not spend too much time on individual items in responding. Respond to each of the items using the scale below to describe your degree of agreement with each item. – I set very high standards for myself. | 1: Strongly disagree  2: Disagree  3: Slightly disagree  4: Neutral  5: Slightly agree  6: Agree  7: Strongly agree | None | Scale | Input |
| Q8_13 | 63 | Numeric | The following items are designed to measure attitudes people have towards themselves, their performance, and toward others. There are no right or wrong answers. Please respons to all of the items. Use your first impression and do not spend too much time on individual items in responding. Respond to each of the items using the scale below to describe your degree of agreement with each item. – I am never satisfied with my accomplishments. | 1: Strongly disagree  2: Disagree  3: Slightly disagree  4: Neutral  5: Slightly agree  6: Agree  7: Strongly agree | None | Scale | Input |
| Q8_14 | 64 | Numeric | The following items are designed to measure attitudes people have towards themselves, their performance, and toward others. There are no right or wrong answers. Please respons to all of the items. Use your first impression and do not spend too much time on individual items in responding. Respond to each of the items using the scale below to describe your degree of agreement with each item. – I expect the best from myself. | 1: Strongly disagree  2: Disagree  3: Slightly disagree  4: Neutral  5: Slightly agree  6: Agree  7: Strongly agree | None | Scale | Input |
| Q8_15 | 65 | Numeric | The following items are designed to measure attitudes people have towards themselves, their performance, and toward others. There are no right or wrong answers. Please respons to all of the items. Use your first impression and do not spend too much time on individual items in responding. Respond to each of the items using the scale below to describe your degree of agreement with each item. – I often worry about not measuring up to my own expectations. | 1: Strongly disagree  2: Disagree  3: Slightly disagree  4: Neutral  5: Slightly agree  6: Agree  7: Strongly agree | None | Scale | Input |
| Q8_16 | 66 | Numeric | The following items are designed to measure attitudes people have towards themselves, their performance, and toward others. There are no right or wrong answers. Please respons to all of the items. Use your first impression and do not spend too much time on individual items in responding. Respond to each of the items using the scale below to describe your degree of agreement with each item. – My performance rarely measures up to my standards. | 1: Strongly disagree  2: Disagree  3: Slightly disagree  4: Neutral  5: Slightly agree  6: Agree  7: Strongly agree | None | Scale | Input |
| Q8_17 | 67 | Numeric | The following items are designed to measure attitudes people have towards themselves, their performance, and toward others. There are no right or wrong answers. Please respons to all of the items. Use your first impression and do not spend too much time on individual items in responding. Respond to each of the items using the scale below to describe your degree of agreement with each item. – I am not satisfied even when I know I have done my best. | 1: Strongly disagree  2: Disagree  3: Slightly disagree  4: Neutral  5: Slightly agree  6: Agree  7: Strongly agree | None | Scale | Input |
| Q8_18 | 68 | Numeric | The following items are designed to measure attitudes people have towards themselves, their performance, and toward others. There are no right or wrong answers. Please respons to all of the items. Use your first impression and do not spend too much time on individual items in responding. Respond to each of the items using the scale below to describe your degree of agreement with each item. – I try to do my best at everything I do. | 1: Strongly disagree  2: Disagree  3: Slightly disagree  4: Neutral  5: Slightly agree  6: Agree  7: Strongly agree | None | Scale | Input |
| Q8_19 | 69 | Numeric | The following items are designed to measure attitudes people have towards themselves, their performance, and toward others. There are no right or wrong answers. Please respons to all of the items. Use your first impression and do not spend too much time on individual items in responding. Respond to each of the items using the scale below to describe your degree of agreement with each item. – I am seldom able to meet my own high standards of performance. | 1: Strongly disagree  2: Disagree  3: Slightly disagree  4: Neutral  5: Slightly agree  6: Agree  7: Strongly agree | None | Scale | Input |
| Q8_20 | 70 | Numeric | The following items are designed to measure attitudes people have towards themselves, their performance, and toward others. There are no right or wrong answers. Please respons to all of the items. Use your first impression and do not spend too much time on individual items in responding. Respond to each of the items using the scale below to describe your degree of agreement with each item. – I am hardly ever satisfied with my performance. | 1: Strongly disagree  2: Disagree  3: Slightly disagree  4: Neutral  5: Slightly agree  6: Agree  7: Strongly agree | None | Scale | Input |
| Q8_21 | 71 | Numeric | The following items are designed to measure attitudes people have towards themselves, their performance, and toward others. There are no right or wrong answers. Please respons to all of the items. Use your first impression and do not spend too much time on individual items in responding. Respond to each of the items using the scale below to describe your degree of agreement with each item. – I hardly ever feel that what I’ve done is good enough. | 1: Strongly disagree  2: Disagree  3: Slightly disagree  4: Neutral  5: Slightly agree  6: Agree  7: Strongly agree | None | Scale | Input |
| Q8_22 | 72 | Numeric | The following items are designed to measure attitudes people have towards themselves, their performance, and toward others. There are no right or wrong answers. Please respons to all of the items. Use your first impression and do not spend too much time on individual items in responding. Respond to each of the items using the scale below to describe your degree of agreement with each item. – I have a strong need to strive for excellence. | 1: Strongly disagree  2: Disagree  3: Slightly disagree  4: Neutral  5: Slightly agree  6: Agree  7: Strongly agree | None | Scale | Input |
| Q8_23 | 73 | Numeric | The following items are designed to measure attitudes people have towards themselves, their performance, and toward others. There are no right or wrong answers. Please respons to all of the items. Use your first impression and do not spend too much time on individual items in responding. Respond to each of the items using the scale below to describe your degree of agreement with each item. – I often feel disappointment after completing a task because I know I could have done better. | 1: Strongly disagree  2: Disagree  3: Slightly disagree  4: Neutral  5: Slightly agree  6: Agree  7: Strongly agree | None | Scale | Input |
| Q9_1 | 74 | Numeric | Please read each below statement and use the scale to indicate the extent to which you agree or disagree with that statement. – I delay making decisions until it’s too late. | 1: Strongly disagree  2: Disagree  3: Somewhat disagree  4: Neither agree nor disagree  5: Somewhat agree  6: Agree  7: Strongly agree | None | Scale | Input |
| Q9_2 | 75 | Numeric | Please read each below statement and use the scale to indicate the extent to which you agree or disagree with that statement. – Even after I make a decision I delay acting upon it. | 1: Strongly disagree  2: Disagree  3: Somewhat disagree  4: Neither agree nor disagree  5: Somewhat agree  6: Agree  7: Strongly agree | None | Scale | Input |
| Q9_3 | 76 | Numeric | Please read each below statement and use the scale to indicate the extent to which you agree or disagree with that statement. – I waste a lot of time on trivial matters before getting to the final decisions. | 1: Strongly disagree  2: Disagree  3: Somewhat disagree  4: Neither agree nor disagree  5: Somewhat agree  6: Agree  7: Strongly agree | None | Scale | Input |
| Q9_4 | 77 | Numeric | Please read each below statement and use the scale to indicate the extent to which you agree or disagree with that statement. – In preparation for some deadlines, I often waste time by doing other things. | 1: Strongly disagree  2: Disagree  3: Somewhat disagree  4: Neither agree nor disagree  5: Somewhat agree  6: Agree  7: Strongly agree | None | Scale | Input |
| Q9_5 | 78 | Numeric | Please read each below statement and use the scale to indicate the extent to which you agree or disagree with that statement. – Even jobs that require little else except sitting down and doing them, I find that they seldom get done for days. | 1: Strongly disagree  2: Disagree  3: Somewhat disagree  4: Neither agree nor disagree  5: Somewhat agree  6: Agree  7: Strongly agree | None | Scale | Input |
| Q9_6 | 79 | Numeric | Please read each below statement and use the scale to indicate the extent to which you agree or disagree with that statement. – I often find myself performing tasks that I had intended to do days before. | 1: Strongly disagree  2: Disagree  3: Somewhat disagree  4: Neither agree nor disagree  5: Somewhat agree  6: Agree  7: Strongly agree | None | Scale | Input |
| Q9_7 | 80 | Numeric | Please read each below statement and use the scale to indicate the extent to which you agree or disagree with that statement. – I am continually saying “I’ll do it tomorrow.” | 1: Strongly disagree  2: Disagree  3: Somewhat disagree  4: Neither agree nor disagree  5: Somewhat agree  6: Agree  7: Strongly agree | None | Scale | Input |
| Q9_8 | 81 | Numeric | Please read each below statement and use the scale to indicate the extent to which you agree or disagree with that statement. – I generally delay before starting on work I have to do. | 1: Strongly disagree  2: Disagree  3: Somewhat disagree  4: Neither agree nor disagree  5: Somewhat agree  6: Agree  7: Strongly agree | None | Scale | Input |
| Q9_9 | 82 | Numeric | Please read each below statement and use the scale to indicate the extent to which you agree or disagree with that statement. – I find myself running out of time. | 1: Strongly disagree  2: Disagree  3: Somewhat disagree  4: Neither agree nor disagree  5: Somewhat agree  6: Agree  7: Strongly agree | None | Scale | Input |
| Q9_10 | 83 | Numeric | Please read each below statement and use the scale to indicate the extent to which you agree or disagree with that statement. – I don’t get things done on time. | 1: Strongly disagree  2: Disagree  3: Somewhat disagree  4: Neither agree nor disagree  5: Somewhat agree  6: Agree  7: Strongly agree | None | Scale | Input |
| Q9_11 | 84 | Numeric | Please read each below statement and use the scale to indicate the extent to which you agree or disagree with that statement. – I am not very good at meeting deadlines. | 1: Strongly disagree  2: Disagree  3: Somewhat disagree  4: Neither agree nor disagree  5: Somewhat agree  6: Agree  7: Strongly agree | None | Scale | Input |
| Q9_12 | 85 | Numeric | Please read each below statement and use the scale to indicate the extent to which you agree or disagree with that statement. – Putting things off till the last minute has cost me money in the past. | 1: Strongly disagree  2: Disagree  3: Somewhat disagree  4: Neither agree nor disagree  5: Somewhat agree  6: Agree  7: Strongly agree | None | Scale | Input |
| FFMQ_Total | 86 | Numeric | Total scores for the 15-item five-facet mindfulness questionnaire (FFMQ-15) | None | None | Scale | Input |
| Acting_with_awareness | 87 | Numeric | Scores for the ‘acting with awareness’ subscale of the FFMQ-15; items 3 (Q6_3), 8 (Q6_8) and 13 (Q6_13) | None | None | Scale | Input |
| Describing | 88 | Numeric | Scores for the ‘describing’ subscale of the FFMQ-15; items 2 (Q6_2), 7 (Q6_7) and 12 (Q6_12) | None | None | Scale | Input |
| Nonjudging | 89 | Numeric | Scores for the ‘nonjudging of inner experience’ subscale of the FFMQ-15; items 4 (Q6_4), 9 (Q6_9) and 14 (Q6_14) | None | None | Scale | Input |
| Nonreactivity | 90 | Numeric | Scores for the ‘nonreactivity to inner experience’ subscale of the FFMQ-15; items 5 (Q6_5), 10 (Q6_10) and 15 (Q6_15) | None | None | Scale | Input |
| Observing | 91 | Numeric | Scores for the ‘observing’ subscale of the FFMQ-15; items 1 (Q6_1), 6 (Q6_6) and 11 (Q6_11) | None | None | Scale | Input |
| ANXIETY | 92 | Numeric | Total scores for the state-trait anxiety inventory-trait form (STAI-T) | None | None | Scale | Input |
| APS_DISCREPANCY | 93 | Numeric | Scores for the ‘discrepancy’ subscale of the almost perfect scale-revised (APS-R); items 3 (Q8_3), 6 (Q8_6), 9 (Q8_9), 11 (Q8_11), 13 (Q8_13), 15 (Q8_15), 16 (Q8_16), 17 (Q8_17), 19 (Q8_19), 20 (Q8_20), 21 (Q8_21) and 23 (Q8_23) | None | None | Scale | Input |
| APS_Standards | 94 | Numeric | Scores for the ‘high standards’ subscale of the APS-R; items 1(Q8_1), 5 (Q8_5), 8 (Q8_8), 12 (Q8_12), 14 (Q8_14), 18 (Q8_18) and 22 (Q8_22) | None | None | Scale | Input |
| APS_Order | 95 | Numeric | Scores for the ‘orderliness’ subscale of the APS-R; items 2 (Q8_2), 4 (Q8_4), 7 (Q8_7) and 10 (Q8_10) | None | None | Scale | Input |
| Perfectionism_Total | 96 | Numeric | Total scores for the APS-R | None | None | Scale | Input |
| PROCRASTINATION | 97 | Numeric | Total scores for the pure procrastination scale (PPS) | None | None | Scale | Input |

Note: Q6_1 to Q6_15 are the items of the 15-item five-facet mindfulness questionnaire (FFMQ-15).

Q7_1 to Q7_20 are the items of the state-trait anxiety inventory-trait form (STAI-T).

Q8_1 to Q8_23 are the items of the almost perfect scale-revised (APS-R).

Q9_1 to Q9_12 are the items of the pure procrastination scale (PPS).
